# The Association of Toll-like Receptor-9 Gene Single-Nucleotide Polymorphism and *AK155(IL-26)* Serum Levels with Chronic Obstructive Pulmonary Disease Exacerbation Risk: A Case-Controlled Study with Bioinformatics Analysis

**DOI:** 10.3390/biomedicines13030613

**Published:** 2025-03-03

**Authors:** Entsar R. Mokhtar, Salwa I. Elshennawy, Heba Elhakeem, Rayyh A. M. Saleh, Sawsan Bakr Elsawy, Khadiga S. M. Salama, Maha Fathy Mohamed, Rania Hamid Bahi, Hayam H. Mansour, Sammar Ahmed Kasim Mahmoud, Marwa M. Hassan, Sara M. Elhadad, Hanaa Mohammed Eid El Sayed, Aliaa N. Mohamed, Nadia M. Hamdy

**Affiliations:** 1 Clinical Pathology Department, Faculty of Medicine (for Girls), Al-Azhar University, Nasr City, Cairo 11884, Egypt; 2 Chest Disease Department, Faculty of Medicine (for Girls), Al-Azhar University, Nasr City, Cairo 11884, Egypt; 3 Chest Disease Department, King Abdul-Aziz Specialized Hospital, Taif 26521, Saudi Arabia; 4 Chest Disease Department, Faculty of Medicine, Beni-Suef University, Beni-Suef 62521, Egypt; 5 Chest Disease Department, Faculty of Medicine, Zagazig University, Zagazig 44519, Egypt; 6 Internal Medicine Department, Faculty of Medicine (for Girls), Al-Azhar University, Nasr City, Cairo 11884, Egypt; 7 Internal Medicine Department, College of Medicine, Taibah University, Al-Madinah P.O. Box 30097, Saudi Arabia; 8 Faculty of Medicine, New Mansoura University, Mansoura 35712, Egypt; 9 Biochemistry Department, Faculty of Pharmacy, Ain Shams University, Abassia, Cairo 11566, Egypt

**Keywords:** chronic obstructive pulmonary disease (COPD), single-nucleotide polymorphism (SNP), toll-like receptor-9 (TLR9), interleukin-26 AK155(IL-26), exacerbation, forced expiratory volume, forced vital capacity, in silico analysis

## Abstract

**Background:** A crucial challenge is the determination of chronic obstructive pulmonary disease (COPD) immune-related mechanisms, where one of the important components of the inflammatory axes in COPD is Toll-like receptor-9 (TLR9) and interleukin-26 AK155(IL-26). Aim: To examine the relation between *TLR9* (T1237C) SNP *rs5743836* and serum levels of AK155(IL-26) with the exacerbation of COPD. Subjects: A total of 96 COPD patients sub-classified into two groups. **Materials:** DNA was purified from blood samples of stable COPD patients (n = 48) vs. exacerbated COPD patients (n = 48) as well as 42 age- and sex-matched healthy smokers and passive smokers as a control group. **Methods:** Genotyping for *TLR9 rs5743836* (T1237C) polymorphism was performed using real time polymerase chain reaction (RT-PCR). AK155(IL-26) serum levels were determined using ELISA. **Results:** There is a significantly higher frequency of the mutant homozygous genotype (C/C) and the mutated C allele of *TLR9 rs5743836* (T1237C) in COPD patients and in the exacerbated group when compared with the control group and stable COPD patients, respectively, with OR 31.98, 1.8 to 57.7, and OR 3.64, 0.98 to 13.36, respectively. For the mutated C allele, the OR was 3.57, 1.94 to 6.56, *p* = 0.001, OR 1.83, 1.02 to 3.27, *p* = 0.041, respectively. In the exacerbated COPD group, there was a significant association between *TLR9* rs5743836 SNP and BMI and the lung vital function measures, CRP, and AK155(IL-26). The exacerbated COPD group has higher serum levels of AK155(IL-26) compared with the stable group or when compared with the control group (*p* = 0.001) for both. AK155(IL-26) serum levels have a positive significant correlation with CRP and BMI and a significant negative correlation with FEV1% and FEV1/FVC in exacerbated COPD patients. **Conclusions:** Our results demonstrated a relation linking *TLR-9 rs5743836* (T1237C) expression and the risk of COPD development and its exacerbation, indicating that dysfunctional polymorphisms of the innate immune genes can affect COPD development and its exacerbation. AK155(IL-26) upregulation was related to decreased lung functionality, systematic inflammatory disease, and COPD exacerbation.

## 1. Introduction

### 1.1. Problem

One of the leading causes of morbidity, mortality, and financial burden worldwide is COPD. Additionally, COPD is one of the third leading causes of death in the world [1]. The dearth of efficient medication(s) results from a misunderstanding of COPD mechanisms and pathophysiology [2].

There is growing evidence that patients with COPD have an accumulation of immune cells in their bronchial biopsy specimens. Air pollution, cigarette smoking, and biomass fuels are significant contributors to the development of COPD [3]. They interfere with the host’s innate defense system by damaging cells via the attack of oxidants, disrupting the epithelial barrier, reducing muco-ciliary clearance, and increasing mucus production [4]. Most exacerbations of COPD are caused by bacterial or viral respiratory infections, which raise airway and systemic inflammation levels [5]. Additionally, genome-wide association studies (GWAS) have demonstrated a substantial association between SNPs and various diseases such as cancer [6], multiple aspects of COPD pathogenesis, and susceptibility to COPD [7].

TLRs, upon activation, play a critical task in triggering innate immunity [8]. There are eleven TLRs, and each recognizes a distinct set of ligand-antigens [9]. The *TLR9* gene is placed on chromosome 3p21.3, approximately 5 kb, with two exons as a coding gene, and its major coding region lies on the second exon. On the promoter region of *TLR9*, the SNP rs5743836 is placed [10]. Studies have shown that the pathogenesis of COPD and emphysema may be influenced by impaired signaling of receptors of the innate immune system.

TLR-9 is one of these receptors that appears to play a crucial role in maintaining lung homeostasis by acting as a pathogen recognition receptor. TLR-9 recognizes viral or bacterial single-stranded (ss) DNA, which results in discriminating between increased percent of un-methylated cytosine-P-guanine dinucleotide (CpG) [11].

TLR9 is located intracellularly [12]. CpG-DNA binding to TLR9 stimulates myeloid differentiation primary response gene 88 (MyD88) release as well as nuclear factor kappa B cell (NF-_k_B) translocation to the nucleus and its activation. All these effects initiate tumor necrosis factor-alpha (TNF-alpha) and interleukin synthesis (IL-6 and IL-1B) [13].

Thus, polymorphisms may affect *TLR9* signaling by decreasing the responsiveness of receptors and increasing susceptibility for bacterial, viral, and fungal infections [14]. Others demonstrated that *TLR9* SNPs were associated with increased TLR9 transcriptional activity, which impairs its signaling [10]. *TLR9* SNP *rs5743836* (T1237C) is related to alveolar macrophages’ dysfunctional innate immune responses and was correlated with COPD severity [15].

Cytokines play an imp. role in chronic inflammation, generally being anti-/pro-inflammatory in various diseases [16] and in COPD [17]. The majority of them can communicate via the Janus kinase (JAK) signal transducer and the signal transducer and activator of transcription (STAT) pathways; they are a result of the signaling pathway JAK-STAT [18].

AK155: Interleukin (IL)-26 has been discovered to trigger JAK1 and tyrosine kinase (TyK) 2 signaling, which results in the phosphorylation and activation of STAT1 and STAT3 [19]. There is compelling evidence that the JAK-STAT pathway, particularly STAT1 and STAT3, which are targets for COPD treatments, is involved in severe COPD stages [20].

AK155(IL-26) is widely expressed in human airways and is believed to be essential for host defense due to its strong anti-bacterial and anti-viral immune responses and neutrophil-mobilizing properties [21]. It contributes to alveolar inflammation in long-term tobacco smokers, whether or not they have COPD. This cytokine is produced by both Th17 cells and human alveolar macrophages [22].

AK155(IL-26) stimulates a neutrophils’ chemotactic response to the inflammatory and bacterial stimuli [23]. Additionally, AK155(IL-26) promotes neutrophil recruitment toward the broncho-alveolar space caused by endotoxin [24]. In long-term smokers, extracellular AK155(IL-26) levels were more expressed in the induced sputum of exacerbated COPD patients when compared with stable cases [25].

Interestingly, a significant gradual increase in AK155(IL-26) levels in the sputum was observed in the period just before an exacerbation; this increase occurred 17 days prior to the clinical attack, indicating that AK155(IL-26) has a potential role as a biomarker of upcoming exacerbations [25,26]. These data demonstrate the essential roles of TLR-9 and AK155(IL-26) in COPD pathogenesis.

### 1.2. Study Aim

We conducted a case-controlled study of 138 individuals to investigate the association of one *TLR-9* SNP and the serum levels of AK155(IL-26) with the exacerbation of COPD in Egyptian patients’ cohort. We examined the relationship of this SNP to the risk of COPD development and progression. It is noteworthy to mention that this is the first research that tested the relationship between *TLR9* gene polymorphism (rs5743836) and serum levels of AK155(IL-26) with the exacerbation of COPD.

## 2. Subjects and Methods

### 2.1. Study Design

This is an observational age and sex-controlled mono-center study, which was conducted between February 2023 to January 2024.

### 2.2. Sample Size Calculation

The sample size was calculated by Epi info, Atlanta, Georgia (US) according to the annual flow of COPD cases and its prevalence in Egypt 6.6 [27], with a margin of error of 5% and confidence level of 95%, and it was found in 96 COPD patients.

### 2.3. Study Participants

A total of 96 patients with COPD (48 stable and 48 exacerbated cases) were enrolled from the Chest Department, Al-Zahraa University Hospital, Faculty of Medicine for Girls, Al-Azhar University. Comparison was performed with 48 age- and sex-matched apparently healthy individuals with normal lung function as a control group.

Patients’ Inclusion and Exclusion criteria:

Full medical history and complete clinical examination were performed for all participants and recorded. Comprehensive chest examination was performed for all participants to ensure their eligibility for enrollment in this study after notifying them and signing the informed consent. Smoking amount, throughout life, was determined as the smoking index (SI) (number of packs of 20 cigarettes per day multiplied by years of smoking). The body mass in kilograms divided by square height in meters (kg/m^2^) was recorded as the body mass index (BMI). COPD patients included male and female individuals more than 18 years old with a diagnosis of COPD. COPD diagnosis was assessed via pulmonary function examination according to GOLD 2024 guideline (Global Initiative for Obstructive Lung Disease) [28]. COPD severity was determined based on Spiro-metric findings, which were performed for all patients and controls as per the American Thoracic Society and European Respiratory Society guidelines [29]. COPD patients taking any medications were recorded.

The exclusion criteria included age below 18 years or more than 90 years; recent pulmonary infections; previous diagnosis of any other chronic lung diseases (obstructive sleep apnea syndrome, interstitial lung disease, and asthma) or any other acute pulmonary disease, such as pulmonary fibrosis and tuberculosis or another acute or chronic heart disease (such as acute coronary syndrome, congestive heart failure, recent revascularization, or unstable angina); recent thoracic surgery; oral anticoagulant therapy; neoplasia; kidney failure; allergies; active or history of autoimmune diseases; and genetic disorders.

### 2.4. Blood Sample Collection

Venous blood samples of about 6 mL were obtained from all participants using standard antecubital venipuncture method after overnight fasting and divided into three portions. First, two ml was evacuated into sterile EDTA-coated tubes, and whole blood was preserved at −20 °C before further processing for genotyping of TLR9 *rs5743836* using PCR. Second, two ml was evacuated into EDTA vacutainer tubes, which were used for CBC determination using Cell-dyn Ruby (Abbott GmbH & Co. KG. Max-Planck-Ring 2. 65205 Wiesbaden, Germany). Third, two ml of blood was evacuated into plain tubes without anticoagulants, then centrifuged at 4000 rpm for fifteen minutes at 20–25 °C. Sera were collected and used for CRP determination (using Cobas C311). The remaining serum was stored at −20 °C until ELISA assay was performed for measurement of AK155(IL-26). Syringes with heparin were used for collection of arterial blood samples from all subjects for ABG assays (using ABG Gem Premier-300).

#### 2.4.1. Molecular Analysis

##### DNA Extraction and Genotyping

Whole blood samples were used for genomic DNA extraction using Blood Genomic DNA Purification Mini Kit (Lot. No. 01198940), Thermo-Fisher Scientific Gene JET Whole Blood Genomic DNA Purification Mini Kit, Baltics UAB V. A. Graiciuno 8, LT-02241 Vilnius, Lithuania), according to the manufacturer’s instructions.

##### Assessment of Genomic DNA Concentration and Purity

DNA samples were assessed for concentration and quality using the QIA Expert spectrophotometer (Qiagen), stored at −20 °C.

DNA concentrations were assessed by recording the absorbance at 260 nm (A260) and at 280 nm (A280), respectively. The two absorbance ratios at 260 and 280 nm were used to determine DNA purity. A “pure” DNA accepted ratio was ~1.8. A lower ratio (≤1.6) may refer to the presence of phenol, proteins, or other contaminants

A260 of 1.0 = 50 μg/mL pure dsDNA (DNA conc. μg/mL) = (A_260_–A_320_ readings) × dilution factor × 50 μg/mL

##### TLR9 SNP TaqMan Genotyping Assay

PCR consisted of 25–50 cycles performed in a 20 μL reaction volume, including 10 uL of TaqMan Genotyping Master Mix (Lot. No. 01187540), 9.5 µL of diluted DNA template (5.5 μL nuclease-free water, Lot No. 01069419, and 4 μL template), and 0.5 µL of TaqMan Genotyping Assay Mix. Data were collected after amplification, and fluorescence signals were determined using a real-time system Rotor Gene from QIAGEN, Hilden, Germany. Every TaqMan^®^ SNP Genotyping Assay contains specific sequence for forward and reverse primers for amplification of the polymorphic sequence of interest and TaqMan^®^ minor groove binder (MGB) probes with non-fluorescent quenchers (NFQ): one probe was labeled with VICTM dye to assess the sequence of allele 1 and another probe was labeled with FAMTM dye to determine 2 sequences of the allele. Context Sequence [VIC/FAM] for TLR9 (rs5743836) is TGGCCATTGTTATTTTTGTTTTACA[G/C]CTGAAGAAACTGAGACTCCATAAGT).

The TaqMan^®^ SNP Genotyping Assay of *TLR9* rs5743836 was obtained from Applied Biosystems by Thermo Scientific, Waltham, MA, USA (Lot No. P211222-005A11).

The discrimination data of alleles were plotted as a comparison of allele 1 (VICTM dye) and allele 2 (FAMTM dye) by using real-time PCR instrument software (QIAGEN, Hilden, Germany). Every specimen is represented as a separate point on the allelic discrimination plot, sometimes referred to as a scatter plot cluster.

##### Quantification of Serum AK155(IL-26)

Serum AK155(IL-26) levels were quantified using a commercially available human ELISA kit (Bioassay Technology Laboratory, Shanghai, China, (cat. No. E0053Hu, lot No. 202212014)). The sensitivity of the assay was 0.23 ng/L, with a standard curve range of 0.5–60 ng/L, Intra-Assay Precision: CV < 8%, Inter-Assay Precision: CV < 10%. ELISA system included a plate shaker-incubator (Thermo-Shaker from EU for Grant Instruments Ltd., Cambs, UK), a plate reader (AS 1851 from DAS, Palombara Sabina, Italy), and an ELISA washer (EL × 50 Biokit, Rome, Italy). All procedures were performed according to the manufacturer’s instructions.

### 2.5. In Silico Database(s) Search and Bioinformatics Analysis

#### 2.5.1. In Silico Identification of Immune Cells

Uniform Manifold Approximation and Projection (UMAP) was used to visualize closely related immune cells from release of the human peripheral blood mononuclear cell single cells or eye immune cells [12]. This was performed using the Human Universal Single Cell Hub (HUSCH), a scRNA-seq database http://husch.comp-genomics.org/#/info_tissue/ (accessed on 4 September 2023).

#### 2.5.2. PICKLE (Protein InteraCtion KnowLedgebasE)

Ref. [13] Release 3.3, 1 October 2021. http://www.pickle.gr/. A meta-database providing the direct protein–protein interactome of the human proteomes, integrating publicly available source protein–protein interaction (PPI) databases via genetic information ontology. The visualization utilizes Cytoscape.js 3.3.0, Cytoscape Panzoom, Font Awesome 4.0.3, FileSaver.js, Arbor.js 0.91, WebCola, and Cose-Bilkent.js resources. Accessed on 9 November 2023.

#### 2.5.3. Gene–Gene Interactions and Pathways by Bioinformatics Analysis

Prediction of B cells surface antigens CD19/CVID3 and CD27/TNFR top interacting genes via gene-interaction at University of California Santa Cruiz (UCSC) [14] Genome Browser RRID:SCR_005780. Genomics Institute: http://genome.ucsc.edu/index.html. Accessed on 6 September 2023.

### 2.6. Statistical Analysis

SPSS version 23.0 Inc. (Chicago, IL, USA) was used for analysis of recorded data. Kolmogorov–Smirnov and Shapiro–Wilk Tests were used to explore data for normality. Mean ± standard deviation (SD) was used to present quantitative data when their distribution was parametric. Median with inter-quartile range (IQR 25–75 percentiles) were used for non-parametric data presentation. However, qualitative variables were presented as percentages and frequencies. Independent student’s *t*-test was used for comparison between two groups. One-way analysis of variance (ANOVA) test was used for comparison between more than groups, and Post Hoc test (Tukey’s) was used for multiple comparisons between different variables. Chi-square test and Fisher’s exact test were used for comparing groups with qualitative data. Correlations between the investigated parameters were analyzed using the Spearman’s correlation coefficient. MedCalc was used to plot the ROC curve used to predict AK155(IL-26) diagnostic utility, the best cut-off with the highest specificity and sensitivity. To assess odds ratio (OR) of genotypes between groups, logistic regression analysis was used. A *p*-value was significant if less than 0.05.

## 3. Results

### 3.1. Study Participants’ Clinico-Pathological and Demographic Details

COPD patients, controls, and exacerbated and stable COPD groups’ baseline demographics are presented in Table 1. When comparing COPD patients with the control group, there were no statistical discriminations per age, gender, and CBC parameters except for WBCs, platelets, and mean corpuscular hemoglobin concentration (MCHC). Significant differences in terms of BMI, smoking index (SI), spirometry parameters, serum levels of CRP, and ABG parameters except HCO_3_ were found. When comparing exacerbated with stable COPD patients, there were no significant differences in terms of age, sex, smoking index, and CBC parameters, except for WBCs and platelets, but there were differences regarding BMI, spirometry parameters, serum levels of CRP, and ABG parameters (*p* less than 0.05), except for PO_2_.

### 3.2. COPD and Control Groups TLR9 rs5743836

The genotype distribution detailed results are presented in Table 2. The incidence of genotypes of *TLR9 rs5743836* (T1237C) SNP was as follows: the wild genotype (T/T) is expressed in (32.1%) of COPD patients, (66.7%) of the control group, (22.9%) of exacerbated COPD patients, and (41.7% of stable) COPD patients. The mutant heterozygous (T/C) is expressed in (52.1%) of COPD patients, (33.3%) of the control group, (56.3%) of exacerbated COPD patients, and (47.9%) of stable COPD patients. The mutant homozygous (C/C) is expressed in (15.6%) of COPD patients, (0.0%) of the control group, (20.8%) of exacerbated COPD patients, and (10.4%) of stable COPD patients. In COPD patients, there were significant differences in the frequency of the mutant homozygous (C/C) genotype (OR 31.98, 95% CI 1.8 to 57.7, *p* = 0.018) when compared with the control group. This revealed that patients with this genotype were at a 31.9 times increased risk of developing COPD. Additionally, the frequency of mutant heterozygous genotype (T/C) was significantly increased in COPD patients comparable to controls (OR 3.22, 1.5 to 6.82, *p* = 0.002), which revealed that patients with this genotype have a 3.2-fold increased risk of developing COPD.

We further observed more mutated *TLR9 rs5743836* “C” (T1237C) in COPD patients vs. controls (OR 3.57, 1.94 to 6.56, *p* = 0.001).

The examination of various inheritance models for *TLR9* (T1237C) *rs5743836* showed that the dominant one with patients having a mutant *TLR9* (T/C) genotype was related to a significantly higher COPD risk development and was the best inheritance model (OR 4.19, 95% CI 2.01 to 8.76, *p* = 0.001). The recessive model with mutant *TLR9* (C/C) patients also had a greater risk of COPD development (OR 18.45, 1.08 to 35.29, *p* = 0.04).

### 3.3. TLR9 rs5743836 and Disease Severity

There was a significantly higher frequency of the mutant homozygous genotype (C/C) in exacerbated COPD patients when compared with the stable group (OR 3.64, 95% CI 0.98 to 13.36, *p* = 0.05), which revealed that patients with this genotype were at a 3.6 times increased risk of developing COPD exacerbation. However, the frequency of the mutant heterozygous genotype (T/C) shows no significant difference between exacerbated COPD patients and the stable group (OR 2.13 95% CI 0.84 to 5.37, *p* = 0.107), which means that this genotype is not associated with COPD exacerbations risk.

We also observed an increased mutated *TLR9 rs5743836* (T1237C) “C” allele in exacerbated COPD patients compared with the stable group (OR 1.83 95% CI 1.02 to 3.27, *p* = 0.041). This indicated that patients who have this allele were at a 1.8 increased risk of developing COPD exacerbation.

The association of various inheritance models for *TLR9 rs5743836* (T1237C) showed that the dominant subjects with the *TLR-9* mutant (T/C) genotype related to COPD exacerbation risk was the best inheritance model (OR 2.4, 0.99 to 5.82, *p* = 0.05).

### 3.4. Association Between TLR9 rs5743836 Genotypes and Different Patient Clinico-Pathological Parameters (Table 3)

In the exacerbated COPD patients’ group (Table 3A), there was correlation between *TLR9 rs5743836* SNP and BMI, FEV1%, FEV1/FVC, CRP, and AK155(IL-26) (*p* = 0.001) for all. Also, similar data were found in the stable COPD patient group (Table 3B).

**Table 3:** (**A**): Association between *TLR9 rs5743836* SNP and different parameters in the exacerbated COPD patients (n = 48). (**B**): Association between *TLR9 rs5743836* SNP with different parameters in stable COPD patient group (n = 48).

(A)				
	*TLR9 rs5743836* in Exacerbated COPD Patients (n = 48)			Test Value
	TT (n = 11)	TC (n = 27)	CC (n = 10)	
Sex				
Female	1 (9.1%)	10 (37.0%)	1 (10.0%)	4.771
Male	10 (90.9%)	17 (63.0%)	9 (90.0%)	
Age (years)	50.27 ± 5.31	51.74 ± 10.04	49.90 ± 10.27	1.059
SI				
Mild	0 (0.0%)	1 (3.7%)	1 (10.0%)	2.897
Moderate	0 (0.0%)	2 (7.4%)	1 (10.0%)	
Severe	10 (90.9%)	20 (74.1%)	7 (70.0%)	
Passive	1 (9.1%)	4 (14.8%)	1 (10.0%)	
BMI [kg/m^2^]	28.00 ± 5.7C	33.11 ± 2.9B	36.40 ± 0.8A	15.859 *
PH	7.37 ± 0.04	7.37 ± 0.05	7.36 ± 0.05	0.358
PCO_2_ (mmHg)	43.45 ± 9.63	45.52 ± 9.28	47.20 ± 11.18	0.390
PO_2_ (mmHg)	82.36 ± 8.48	82.74 ± 8.53	82.10 ± 10.18	0.021
HCO_3_ (mmol/L)	24.96 ± 1.59	25.07 ± 1.52	25.40 ± 1.78	0.220
S O_2_%	90.36 ± 2.01	90.27 ± 2.58	89.60 ± 2.22	0.342
FEV1%	37.82 ± 5.4A	31.37 ± 4.7B	23.602.4C	26.261 *
FVC%	49.45 ± 8.20	47.37 ± 7.18	50.80 ± 11.01	0.704
FEV1/FVC	67.94 ± 2.3A	64.07 ± 4.0A	59.39 ± 4.8B	12.710 *
VC%	51.82 ± 7.31	50.81 ± 6.40	52.90 ± 10.58	0.288
FEF%	41.18 ± 11.08	40.04 ± 10.86	42.60 ± 6.26	0.240
WBCs (10^3^/mm^3^)	10.08 ± 5.12	10.27 ± 5.04	10.38 ± 5.42	0.009
RBCs (10^3^/mm^3^)	5.23 ± 0.38	5.14 ± 0.46	5.00 ± 0.58	0.638
Hb (gm/dL)	15.34 ± 1.57	14.84 ± 1.56	14.87 ± 2.02	0.364
HCT%	47.98 ± 4.59	46.25 ± 4.93	44.80 ± 5.61	1.070
MCV (µm^3^)	88.52 ± 3.05	87.95 ± 3.20	87.93 ± 2.57	0.150
MCHC (gm/dL)	32.15 ± 1.88	32.67 ± 1.76	32.70 ± 1.06	0.432
PLT (10^3^/mm^3^)	305.82 ± 60.81	308.81 ± 48.12	314.10 ± 43.54	0.073
CRP (gm/L)	8.54 ± 2.23C	10.98 ± 2.05B	17.19 ± 4.86A	25.754 *
AK155(IL-26) (pg/mL)	31.09 ± 5.68C	42.11 ± 4.40B	49.60 ± 1.58A	49.141 *
(**B**)				
	*TLR9 rs5743836* in Stable COPD Patients (n = 48)			Test Value
	TT (n = 11)	TC (n = 27)	CC (n = 10)	
Sex				
Female	2 (10.0%)	5 (21.7%)	1 (20.0%)	1.106
Male	18 (90.0%)	18 (78.3%)	4 (80.0%)	
Age (years)	50.55 ± 11.02	51.78 ± 7.32	50.40 ± 10.57	0.397
SI				
Mild	2 (10.0%)	3 (13.0%)	1 (20.0%)	7.605
Moderate	5 (25.0%)	3 (13.0%)	1 (20.0%)	
Severe	9 (45.0%)	17 (73.9%)	2 (40.0%)	
Passive	4 (20.0%)	0 (0.0%)	1 (20.0%)	
BMI [kg/m^2^]	20.10 ± 1.1C	27.17 ± 3.7B	33.80 ± 2.7A	60.664 *
PH	7.42 ± 0.03	7.41 ± 0.03	7.41 ± 0.02	0.244
PCO_2_ (mmHg)	36.99 ± 1.25	37.64 ± 2.08	37.94 ± 3.41	0.814
PO_2_ (mmHg)	82.10 ± 9.35	83.26 ± 7.31	80.60 ± 13.72	0.217
HCO_3_ (mmol/L)	24.09 ± 1.53	24.22 ± 0.77	23.84 ± 1.41	0.218
S O_2_%	93.10 ± 2.98	93.90 ± 2.40	92.18 ± 1.33	1.128
FEV1%	59.60 ± 8.4A	40.74 ± 7.1B	31.00 ± 6.8C	45.911 *
FVC%	60.93 ± 5.47	59.39 ± 12.84	59.68 ± 6.05	0.135
FEV1/FVC	63.15 ± 8.5A	43.78 ± 6.3B	37.00 ± 3.4C	50.515 *
VC%	24.50 ± 7.78	31.09 ± 10.60	31.00 ± 11.34	2.739
FEF%	30.75 ± 8.87	26.62 ± 8.10	32.33 ± 17.21	0.876
WBCs (10^3^/mm^3^)	6.05 ± 1.13	6.65 ± 1.09	6.44 ± 1.21	1.514
RBCs (10^3^/mm^3^)	5.14 ± 0.37	5.25 ± 0.35	5.18 ± 0.26	0.604
Hb (gm/dL)	13.81 ± 1.21	13.98 ± 0.78	14.98 ± 0.47	2.983
HCT%	44.83 ± 3.00	46.35 ± 3.16	45.36 ± 2.29	1.369
MCV (µm^3^)	88.21 ± 3.23	87.53 ± 6.39	85.56 ± 2.94	0.562
MCHC (gm/dL)	33.47 ± 1.42	32.63 ± 1.32	32.82 ± 1.36	2.022
PLT (10^3^/mm^3^)	292.20 ± 63.13	266.00 ± 61.94	264.60 ± 48.17	1.085
CRP (gm/L)	3.57 ± 0.47	6.27 ± 1.52	8.56 ± 0.57	53.957 *
AK155(IL-26) (pg/mL)	15.21 ± 2.42	25.22 ± 3.69	32.40 ± 1.52	90.995 *

One-way analysis of variance test was performed for Mean ± SD and multiple comparisons between groups through Post Hoc test: Tukey’s test, x^2^: Chi-square test for number (%), or Fisher’s exact test, when appropriate. * A *p*-value < 0.5 is statistically significant. [*TLR9*: Toll-Like Receptor-9, COPD: chronic obstructive pulmonary disease, BMI: body mass index, PCO_2_: Partial Pressure of CO_2_, PO_2_: Partial Pressure of O_2_, HCO_3_: bicarbonate, SI: smoking index, S O_2_%: O_2_ saturation, FEV1%: Forced Expiratory Volume in the first second of expiration, FVC%: Forced Vital Capacity, FEV1/FVC: Forced Expiratory Volume/Forced Vital Capacity, VC%: Vital Capacity, FEF%: Forced Expiratory Flow Rate, WBCs: White Blood Cells, RBCs: Red Blood Cells, Hb: hemoglobin, HCT: hematocrit, MCV: Mean Corpuscular Volume, MCHC: Mean Corpuscular Hemoglobin Concentration, PLT: platelets, CRP: C-Reactive Protein, AK155(IL-26): Interleukin-26, NS: non-significant].

### 3.5. Serum Levels of AK155(IL-26) in COPD Patients, Exacerbated, Stable Groups and Its Correlation with Different Clinical Characteristics

COPD patients and exacerbated groups had a significant increase in the serum levels of AK155(IL-26) compared with the control and stable group, respectively (*p* = 0.001) for both. The serum levels of AK155(IL-26) showed a forward association with CRP and BMI (r = 0.755 and r = 0.790, respectively) and a negative association with FEV1% and FEV1/FVC (r = −0.915 and r = −0.731, respectively) in the exacerbated group (*p* < 0.05). Also, similar data were observed in the stable COPD group, in addition to a positive correlation between AK155(IL-26) and VC%, as shown in Table 4. However, there is no statistically significant association between sex and AK155(IL-26) in each group (*p* > 0.05).

### 3.6. Diagnostic/Prognostic Utility of AK155(IL-26)

**Figure 1:**
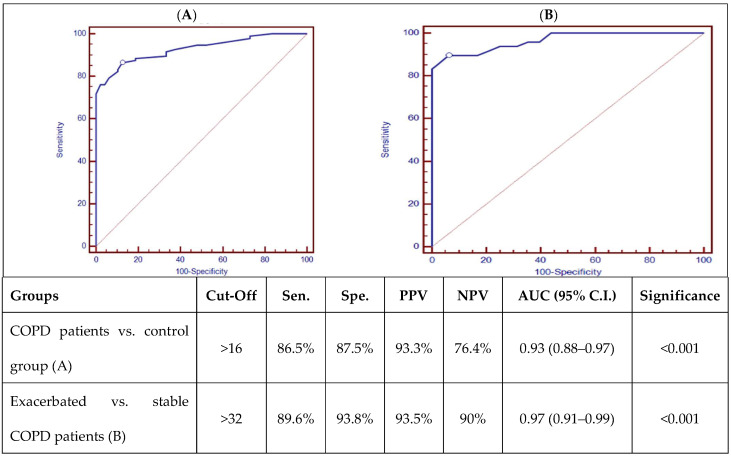
ROC curve analysis of AK155(IL-26) (pg/mL) between COPD patients and control group (**A**) and exacerbated and stable COPD patients (**B**). [Sens.: sensitivity; Spec.: specificity; PPV: positive predictive value; NPV: negative predictive value; AUD: Area Under the Curve; *p* < 0.001 is highly significant].

ROC curve analysis of AK155(IL-26) is presented in Figure 1, which showed good discriminating power between COPD patients and the control group, as well as good discriminating power between stable and exacerbated COPD patients, with a *p*-value (*p* < 0.001).

### 3.7. In Silico Data Processing and Bioinformatics Analysis Accessed 5 November 2024

#### 3.7.1. Immune Cell Infiltration Functional Enrichment Analysis

**Figure 2:**
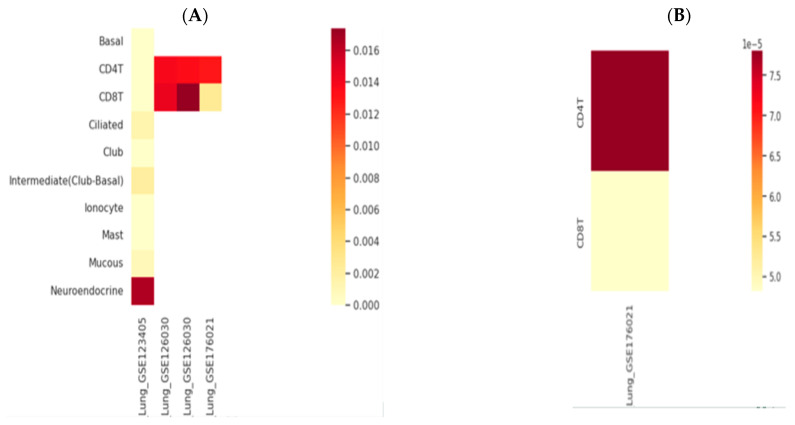
Genes’ relative expression heatmap: (**A**) *AK155(IL-26)* and (**B**) *TLR9*. Legend is 0.0., with yellowish (low expression) through red to dark red color indicating highly expressed gene. http://husch.comp-genomics.org/#/info_tissue/Lung (accessed on 5 November 2024).

*AK155(IL-26)* and *TLR9* genes expression was retrieved through the human lung immune cells, single cells, using an scRNA-seq database; HUSCH applying several datasets http://husch.comp-genomics.org/#/ (accessed on 5 November 2024) search appears in Figure 2. Accessed on 5 November 2024, retrieved from the respiratory system; exploration within lung tissue http://husch.comp-genomics.org/#/info_tissue/Lung (accessed on 5 November 2024) for *AK155(IL-26)* and *TLR9* genes.

#### 3.7.2. Gene-to-Protein, Gene-to-Gene, or Protein-to-Protein Interaction (PPI)

Analyzing signaling pathways and more via SignaLink3.0 v3.1 http://signalink.org/ [30], Accessed on November 2024.

IL26 http://signalink.org/node/Q9NPH9 (accessed on 5 November 2024) is a ligand; it has 11 transcriptional regulators: ETS1, GATA2, TFAP2C, FOXP3, SMARCA4, CEBPA, CTCF, E2F1, EGR1, FOXA1, and RUNX1. The pathway members are presented in the figure below.

TLR9 http://signalink.org/node/Q9NR96 (accessed on 5 November 2024) is a protein, with 25 pathway members, namely, BTK, TLR8, AGER, MYD88, HMGB1, BCL6, CD14, LYAR, KRT85, ELOVL1, CAPZA2, CREB1, ELK1, KRT82, KRT36, ELOF1, RNF216, ACTR3B, ERC1, KRT31, CAPZA1, CAPZB, VRK3, and ETS2.

There are 15 TLR9 transcriptional regulators: ETS1, YBX1, SMARCA4, CTCF, EGR1, FOS, CEBPA, CREB1, ELF1, ELK1, ETS2, HDAC3, NFKB1, PTMA, and RELA.

A Venn diagram was drawn using Venny2.1 [31], revealing cross-regulatory transcription factors among both https://bioinfogp.cnb.csic.es/tools/venny/index.html (accessed on 5 November 2024).

There were five common regulators between AK155(IL-26) “List 1” and TLR-9 “List 2”: ETS1, SMARCA4, CEBPA, CTCF, and EGR1. There is a direct link between AK155(IL-26) and TLR-9, which was evidenced using STRING v12.0.

**Figure 3:**
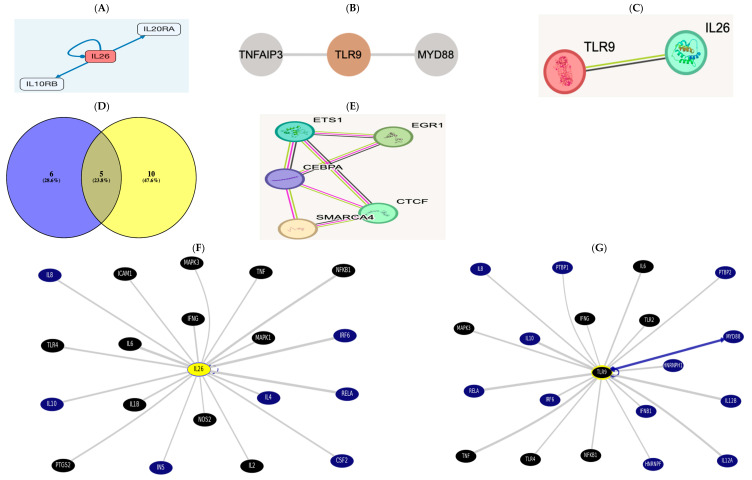
(**A**) The receptor combination of IL10RB and IL20RA is unique and specific for AK155(IL26), presented as pathway members https://omim.org/entry/605679?search=il26&highlight=il26 and http://signalink.org/node/Q9NPH9, as well as https://signor.uniroma2.it/relation_result.php?id=Q9NPH9. (**B**) https://www.proteinatlas.org/ENSG00000239732-TLR9/structure+interaction#interaction and according to https://signor.uniroma2.it/relation_result.php?id=Q9NR96 TLR9 activates myeloid differentiation primary protein 88 (MyD88) and a Toll/Interleukin receptor protein domain. (**C**) The direct link between AK155(IL-26) and TLR-9 evidenced via the STRING version 12.0 database https://string-db.org/cgi/network?taskId=b6gWhpi8e53F&sessionId=b4XNW7ObLS7n. (**D**) List 1, with violet color to the left, is AK155(IL-26), and list 2 indicates TLR9, yellow-colored to the right, showing 5 common regulators between AK155(IL-26) as “List 1” and TLR-9 as “List 2,” which are ETS1, SMARCA4, CEBPA, CTCF, and EGR1. This result is evident when interacting via the STRING, as well as protein interaction via STITCH database. (**E**,**F**) https://genome.ucsc.edu/cgi-bin/hgGeneGraph?gene=IL26&1=OK&supportLevel=text&hideIndirect=on&geneCount=20&geneAnnot=drugbank&1=OK&geneCount=20. (**G**) https://genome.ucsc.edu/cgi-bin/hgGeneGraph?gene=TLR9&1=OK&supportLevel=text&hideIndirect=on&geneCount=20&geneCount=15&geneAnnot=drugbank&1=OK (all accessed on 5 November 2024) [black-colored genes denote treatment hits by Drug Bank; continuous gray line for results indicates dataset interaction curated from source document, and no text-mining line indicates no curated information where text mining is evident; blue continuous line indicates interaction from several datasets with text mining]. Accessed on 5 November 2024.

In the UCSC https://genome.ucsc.edu/cgi-bin/hgGeneGraph?gene, the top interacting genes were both AK155(IL-26) and TLR9, which were targeted using drug bank therapy, as presented in Figure 3F,G, where AK155(IL-26) and TLR9’s top interacting genes(and their target drugs) were IL-6 (Ginseng), TNF (chloroquine and glucosamine), TLR4 (naloxone), NFkB1 (Aspirin), MAPK1 and 3 (Sulindac), and IFNG, targeted by glucosamine. However, AK155(IL-26)’s only top interacting genes were IL-1B, IL-2, ICAM1 (Natalizumab), and NOS_2_ (Dexamethasone and Miconazole), accessed on 5 November 2024.

## 4. Discussion

COPD is a heterogeneous disease, and the immune inflammatory response is believed to play a pivotal role in its pathogenesis [32].

In The Human Protein Atlas from HPA datasets https://www.proteinatlas.org/ AK155, AK155(IL-26) is not a prognostic marker related to the inflammatory response. the AK155(IL-26) gene is located on chromosome 12, cytoband q15, and not detected in lung related diseases cases. The single-cell expression cluster was performed for the immune system according to the Human Protein Atlas from HPA datasets https://www.proteinatlas.org/ENSG00000111536-IL26 (accessed on 5 November 2024).

AK155(IL-26) has a pro-inflammatory function and plays a role in the local mechanisms of immunity and activating MAPK1/3 (ERK1/2), STAT1 and STAT3, and AKT and JUN, with induced expression of SOCS3, IL-8, and TNF-alpha; secretion of IL-10 and IL-8; and surface expression of ICAM1 https://omim.org/entry/605679?search=il26&highlight=il26 (accessed on 5 November 2024).

Per The Human Protein Atlas, TLR9; CD289 is a CD marker and approved FDA drug target, located on chromosome 3, cytoband p21.2. It is not prognostic and not a cancer-enhanced protein confined to the immune system. TLR9 controls the host immune response. TLR9 is a nucleotide-sensing TLR that is activated by unmethylated CpG dinucleotides. It acts via MYD88’s innate immune signal transduction adaptor and TRAF6, leading to NF-kappa-B activation, cytokine secretion, and inflammatory response. Upon CpG stimulation, it induces B-cell proliferation, activation, survival, and antibody production https://www.proteinatlas.org/ENSG00000239732-TLR9 (accessed on 5 November 2024).

TLR9, a pathogen recognition receptor, is the only TLR in humans that can recognize viral or bacterial ss DNA, distinguishing between higher percentages of unmethylated CpG dinucleotides [33]. It is significantly expressed in a variety of lung cell types, including alveolar macrophages, alveolar septae cells, the bronchial epithelium, and vascular endothelium. It plays a pivotal role in COPD pathogenesis [34].

In our study, we investigated the association between *TLR9 rs5743836* T1237C SNP and the risk of COPD and its exacerbation, where we found that the TLR9 *rs5743836* mutant (C/C) genotype and the C allele were expressed in higher frequency in COPD patients and in the exacerbated group compared with control and stable groups, respectively. This means that this SNP is associated with an increased risk for COPD and its exacerbation. We also found that the expression of *TLR9 rs5743836* SNP was associated with FEV1%, FEV/FVC, BMI, and CRP in exacerbated COPD patients. Our results were partially in agreement with the work by Berenson et al. [15], who found that TLR9 *rs5743836* (T1237C) was expressed in higher frequency in active smokers COPD patients compared with COPD ex-smokers and healthy control. Additionally, IL-8 responsiveness to *S. pneumoniae*, *M. catarrhalis*, and *H. influenzae* was strongly associated with the carriage of TLR9 (T1237C) but not with *TLR9* (T1486C) among ex-smoking COPD patients. FEV1% was significantly decreased among *TLR9* (T1237C) SNP COPD patients. They demonstrated that *TLR9* (T1237C) expression is significantly associated with dysfunctional innate alveolar macrophage responses against respiratory pathogens, which was also associated with COPD severity.

Similar findings were observed when analyzing exacerbated COPD (ECOPD) patients and the control group in a study by Alhabeeb et al. [35] where they found (C/C) and (T/C) genotypes and the allele C of TLR9 (T1237C) gene polymorphism were associated with a higher susceptibility risk for COPD. Our findings were partially in accordance with this study, where we found that the *TLR9 rs5743836* mutant (C/C) genotype and the C allele were expressed in higher frequency in exacerbated COPD patients in comparison with the stable group; therefore, this SNP is associated with a higher risk of exacerbation in COPD patients, but our comparison for the genotype (T/C) showed no significant differences between the two groups.

Foronjy et al. [36] demonstrated that DNA viruses such as Epstein–Barr and herpes are detected frequently in COPD patients’ lungs, and through the modulation of TLR9 signaling, they could contribute to disease exacerbations.

Interestingly, among the European–American population, *TLR9 rs5743836* (T1237C) was closely associated with bronchial asthma [37] and an increased prevalence of *TLR9* (T1237C) in allergic bronchopulmonary aspergillosis [38]. TLR9 *rs5743836* (T1237C) expression is also associated with a higher risk of renal disease and premalignant gastric disease induced by H. pylori [39,40]. Interactions may result in an abnormal immune response, as in Crohn’s disease, if the expression of particular TLR SNPs disrupts receptor-ligand contact [41].

We found a higher frequency of the *TLR9* rs5743836 C allele in COPD patients and the exacerbated group in comparison with control and stable groups, respectively. The frequency of this C allele was associated with decreased FEV1% in exacerbated COPD patients. This association suggested the inability of these patients to react strongly to respiratory pathogens via inflammatory response [42].

However, a functional analysis study of SNP demonstrated that C allele carriage was associated with increased transcriptional activity of TLR9, primarily due to NF-kappa B activation [43]. In COPD, the disease site exhibits greater NF-kappa B expression [44].

In compliance with our data, two research studies have investigated the *TLR9* T-1486C SNP’s functionality, and both of them reported that the C-allele of *TLR9* T1486C SNP is linked to a lower expression of receptor under basal conditions [45,46].

In contrast to our results, Pabst et al. [47] found no significant differences for *TLR9 rs5743836* (T1237C) SNP when comparing COPD patients with controls and exacerbated stable cases. Additionally, Lazarus et al. [48] could not find any relation between *TLR9* (T1237C) SNP and COPD development risk. The discrepancies between these results may attributed to genetic background differences and variability in gene frequency between individuals from different countries.

In our study, we found a higher significant increase in the serum levels of AK155(IL-26) in COPD patients in comparison with the control group and exacerbated COPD patients in comparison with the stable group; this result matched findings by Lundgren et al. [49] who showed similar results. Also, Savchenko et al. [50] examined the induced sputum in a group of stable COPD patients and found a significant elevation in AK155(IL-26) levels when comparing them with healthy subjects. This could be explained by Zuo et al. [51] who showed increased numbers of macrophages, neutrophils, and CD8+ T cells in COPD patients’ airways.

Moreover, TLR9 elicits an inflammatory response via initiating neutrophil recruitment, playing an essential role in activated CD4+ T cell proliferation [47]. Moreover, Nadigel et al. [43] demonstrated that COPD patients have abnormal expression of *TLR-9* in lung CD8+ T cells. The expression level of TLR9 may be deregulated due to genetic variations arising from SNP in the *TLR-9* gene, which is able to alter the COPD course or raise disease susceptibility. Moreover, *TLR9* SNPs may upregulate cytokines, chemokines, and signaling molecules in the lung [35]. These were evident in Figure 2 heatmaps.

AK155(IL-26), according to studies, can bind DNA that has been released from injured cells and plays as a carrier molecule for extracellular DNA, further contributing to binding in the inflammation site. According to this method of action, AK155(IL-26) may operate as a trigger and a driver for the inflammatory response, resulting in the establishment of a harmful amplification loop and persistent, chronic inflammation [52].

In addition, we found that serum levels of AK155(IL-26) have a negative correlation with FEV1% and FEV/FVC and a positive correlation with CRP and BMI in exacerbated and stable COPD patients; these matched results found by Savchenko et al. [50] who showed that AK155(IL-26) levels in sputum have a positive correlation with CRP, BMI, and leptin and have a negative correlation with FEV1% and FEV/FVC. Also, AK155(IL-26) levels were increased in obese COPD patients compared with healthy controls. They proposed AK155(IL-26) as a potential marker for determining the level of inflammation in COPD patients’ lung tissue. The signal transduction cascade that follows TLR activation is well established to function through many pathways, such as NF-kB and JNK, which then attach to target DNA sequences to trigger the production of cytokines [53]. Our study demonstrated a strong association between *TLR-9* rs*5743836* SNP and the serum level of AK155(IL-26) in COPD patients. Additionally, Phipps et al. 2010 [54] discovered that exposure to cigarette smoke causes CD8+ cells to express TLR9 and TLR4, which raises cytokine production. Thus, one way that CD8+ T cells can contribute to the pathophysiology of COPD is by the activation of TLRs on CD8+ cells caused by cigarette smoke, which, in turn, increases the production of cytokines.

T_H_17 cell-derived AK155(IL-26) formed complexes with bacterial DNA and self-DNA released via dying bacteria and host cells. The resulting AK155(IL-26)–DNA complexes induced plasmacytoid dendritic cell (PDC) production of interferon type I via the activation of TLR9 but independently of the AK155(IL-26) receptor. These findings provide insights into the potent proinflammatory and antimicrobial function of T_H_17 cells by showing that AK155(IL-26) is a natural human antimicrobial that promotes immune sensing of bacterial and host cell death [55]. AK155(IL-26) produced from T_H_17 cells formed complexes with self-DNA released by dying bacteria and host cells as well as bacterial DNA. By activating TLR9, the resultant AK155(IL-26)–DNA complexes cause plasmacytoid dendritic cells (PDCs) to produce interferon type I without the aid of the AK155(IL-26) receptor. By demonstrating that AK155 (IL-26) is a naturally occurring human antimicrobial that stimulates immunological sensing of bacterial and host cell death, these results provide light on the strong proinflammatory and antimicrobial function of TH17 cells [55].

High levels of AK155(IL-26) expression are seen in autoimmune disorders mediated by T_H_17 cells. High PDC counts and elevated IFN-α expression in tissues are hallmarks of these disorders, indicating that the overexpression of AK155(IL-26) causes unchecked inflammatory responses through heightened IFN-α induction [56].

In confirmation of our data, Cardenas et al. in 2024 [21] demonstrated previously that different structural and immunological cells isolated from human airways produce AK155(IL-26) in response to stimulation of TLR9, which is implicated in the innate recognition of SARS-CoV-2.

According to gene ontology (GO) via PICKEL, http://www.pickle.gr/ regarding AK155(IL-26) and TLR9 indicate cell–cell signaling, cytokine-mediated signaling pathway, positive regulation of stress-activated MAPK cascade and transcription by RNA polymerase II, protein kinase B signaling via JAK-STAT, and negative regulation of epithelial cell and IL signaling/proliferation http://www.pickle.gr/Entity/EntityInfo?pickleid=86494&org=9606 (accessed on 5 November 2024).

Up till now, no drugs for the AK155(IL-26) gene have been available on The Human Gene Database Gene Cards.

https://www.genecards.org/cgi-bin/carddisp.pl?gene=IL26&keywords=il26#drugs_compounds (accessed on 5 November 2024), and from the BioGRID database, https://thebiogrid.org/120913/summary/homo-sapiens/il26.html.

As demonstrated by the in silico analysis, AK155(IL-26) blockade targeting may offer therapeutic benefits for COPD treatment through TLR9 suppression for TLR9.

https://www.genecards.org/cgi-bin/carddisp.pl?gene=TLR9&keywords=TLR9#compounds-drugs (accessed on 5 November 2024) and https://thebiogrid.org/119902/summary/homo-sapiens/tlr9.html (accessed on 5 November 2024). Available interacting chemicals and/or drugs for TLR9 are chloroquine and hydroxychloroquine, which is a 4-aminoquinoline with immunosuppressive and anti-autophagy small molecule drugs; Cetuximab, which is an EGFR inhibitor, monoclonal antibody, TOPO1 inhibitor, BRAF inhibitor, ERBB2 inhibitor, MTOR inhibitor, and antineoplastic agent; as well as NO, adenine, and tyrosine.

## 5. Conclusions

The *TLR-9 rs5743836* gene polymorphism can result in clinico-immunological changes related to the severity and susceptibility of COPD. Therefore, individuals with higher-risk genotypes could be identified to receive the available treatments targeting the TLR-9 pathway to minimize the severity of the disease.

AK155(IL-26) overexpression has been linked to decreased lung function, increased systemic inflammation, and impending exacerbated risk events. This suggests that AK155(IL-26) has a strong role in COPD monitoring and is a biomarker of the diagnosis and progression course of the disease. It has been demonstrated that either AK155(IL-26) or TLR9 are independent risk factors for COPD progression.

Mechanistically, AK155(IL-26) binds to its dimeric ILs/IL-receptor comprising IL-20RA and IL-10RB, resulting in the phosphorylation of STAT3 mediated by JAK1 and STAT2-IL [57]. STAT3 then makes its way to the nucleus, inducing the expression of inflammatory mediators to enhance more inflammation and cytokine release. Therefore, a more profound pro-inflammation or acute phase response is there. This will exacerbate COPD adverse events via the *TLR9* mutation–AK155(IL-26) axis.

This current study provided evidence of a connection between AK155(IL-26) and TLR9 *rs5743836* in the COPD-tested cohort cases (Graphical Abstract), which paves the way for targeted immunotherapy alone or with immune-modulatory supplement(s) [58].

Based on the current results and highlighted studies, we can speculate that the investigation of the *TLR9* mutation(s) and their relation to the AK155(IL-26) cytokine have shed light on how to prevent/treat COPD exacerbation. Based on the above in silico studies, polymorphisms of TLRs may be a potential therapeutic target for COPD.

Now, we can suggest that the “COPD Pathogenesis Hallmark(s)” involves a “Molecular-Network being Inflammation-Associated” (retrieved in silico in Figure 3) triggered by reduced lung function and the systemic low-grade inflammation state as an activating stimulus (AK155(IL-26) and increased serum level of CRP). However, the airway microenvironment dysregulation was not currently addressed (to be conducted shortly as work on sustainability).

The limitation of our study was that one SNP in *TLR9* was analyzed, rendering the current study an exploratory case-controlled study. Second, this study did not involve the survival predictive role of the investigated inflammatory as well as the clinicopathological markers and the examined TLR9 SNP.

## Figures and Tables

**Table 1:** Comparison between COPD patients and control groups and stable and exacerbated COPD patients regarding demographic and laboratory data.

	Groups (n)			COPD Group (n)		
Parameters	COPD (96)	Control (48)	*p*-Value	Stable (n = 48)	**Exacerbation (48)**	***p*-Value**
Sex						
Female	20 (20.8%)	13 (27.1%)	NS	8 (16.7%)	12 (25.0%)	NS
Male	76 (79.2%)	35 (72.9%)		40 (83.3%)	36 (75.0%)	
Age (years)						
Mean ± SD	50.68 ± 10.12	49.19 ± 7.80	NS	48.50 ± 9.24	51.85 ± 9.28	NS
Range	36–84	38–70		36–75	42–84	
Exacerbation Severity; n (%)						
Mild/Moderate/Sever	–	–	–	(22,11,15) (45.8%, 22.9%, 31.3%)	(18/12/18) (37.5%/25%/37.5%)	–
Treatment; n (%)						
Long-acting B2 agonist (LABA)				22 (45.8%)	–	
LABA + long-acting muscarinic receptor antagonist (LAMA) + inhaled corticosteroids (ICS) + Azithromycin				11 (22.9%)	–	
LABA + LAMA + ICS+ Roflumilast				15 (31.25%)	–	
Short-acting B2 agonist (SABA) + short–acting muscarinic receptor antagonist (SAMA) + ICS+ Levofloxacin				–	8 (16.7%)	
SABA + SAMA + ICS + Ceftriaxone				–	10 (20.8%)	
SABA + SAMA + Oral Corticosteroids				–	12 (25%)	
SABA + SAMA + Oral Oxygen				–	9 (18.75)	
SABA + SAMA + IV Corticosteroids + Oral Oxygen + Non-invasive mechanical Ventilation (NIV)				–	9 (18.75)	
Smoking index (SI)						
Mild	8 (8.3%)	9 (18.8%)	0.001	6 (12.5%)	2 (4.2%)	NS
Moderate	12 (12.5%)	3 (6.3%)		9 (18.8%)	3 (6.3%)	
Severe	65 (67.7%)	6 (12.5%)		28 (58.3%)	37 (77.1%)	
Passive	11 (11.5%)	30 (62.5%)		5 (10.4%)	6 (12.5%)	
BMI [kg/m^2^]						
Mean ± SD	28.77 ± 6.23	24.46 ± 1.76	0.001	24.92 ± 5.33	32.63 ± 4.45	0.001
Range	18–38	21–28		18–36	20–38	
PH						
Mean ± SD	7.39 ± 0.04	7.42 ± 0.02	0.001	7.41 ± 0.03	7.37 ± 0.05	0.001
Range	7.28–7.47	7.4–7.45		7.36–7.47	7.28–7.43	
PCO_2_ (mmHg)						
Mean ± SD	41.40 ± 8.00	38.92 ± 2.53	0.038	37.40 ± 1.95	45.40 ± 9.64	0.001
Range	33.7–68	35–43		33.7–43	36–68	
PO_2_ (mmHg)						
Mean ± SD	82.51 ± 8.69	87.06 ± 2.79	0.001	82.50 ± 8.78	82.52 ± 8.69	NS
Range	57–93	83–92		57–92	65–93	
HCO_3_ (mmol/L)						
Mean ± SD	24.62 ± 1.47	24.19 ± 0.81	NS	24.12 ± 1.19	25.12 ± 1.56	0.001
Range	21.9–29	23.1–25.5		21.9–26.9	22–29	
S O_2_%						
Mean ± SD	91.77 ± 2.96	97.23 ± 1.46	0.001	93.39 ± 2.60	90.15 ± 2.36	0.001
Range	86–98	95–99		89–98	86–95	
FEV1%						
Mean ± SD	39.41 ± 13.12	83.25 ± 2.84	0.001	47.58 ± 13.01	31.23 ± 6.47	0.001
Range	20–75	80–88		20–75	20–50	
FVC%						
Mean ± SD	54.31 ± 10.63	79.90 ± 1.64	0.001	60.06 ± 9.64	48.56 ± 8.25	0.001
Range	30–86.6	77–83		30–86.6	31–72	
FEV1/FVC						
Mean ± SD	57.56 ± 11.44	86.17 ± 3.22	0.001	51.14 ± 12.56	63.98 ± 4.75	0.001
Range	34–76	83–91		34–76	54–69.4	
VC%						
Mean ± SD	39.91 ± 14.56	86.19 ± 2.22	0.001	28.33 ± 6.94	51.48 ± 7.49	0.001
Range	14–75	83–89		14–53	35–75	
FEF%						
Mean ± SD	36.19 ± 11.25	29.40 ± 6.69	0.001	29.22 ± 7.36	40.83 ± 10.00	0.001
Range	13–55	20–40		19–52	13–55	
WBCs (10^3^/mm^3^)						
Mean ± SD	8.32 ± 4.11	6.40 ± 1.23	0.002	6.38 ± 1.13	10.25 ± 2.78	0.001
Range	4.32–19	4.65–9.5		4.32–8.2	4.8–19	
RBCs (10^3^/mm^3^)						
Mean ± SD	5.16 ± 0.41	5.17 ± 0.31	NS	5.20 ± 0.35	5.13 ± 0.47	NS
Range	4.1–5.9	4.5–5.6		4.5–5.9	4.1–5.8	
Hb (gm/dL)						
Mean ± SD	14.15 ± 10.14	13.54 ± 0.79	NS	14.01 ± 1.05	14.96 ± 1.64	NS
Range	11.4–15.5	11.9–14.8		11.9–15.5	11.4–18	
HCT%						
Mean ± SD	45.98 ± 4.14	44.90 ± 1.27	NS	45.61 ± 3.05	46.35 ± 5.01	NS
Range	35.7–55	42–47.5		35.7–51	37–55	
MCV (µm^3^)						
Mean ± SD	87.84 ± 4.09	86.78 ± 3.65	NS	87.60 ± 4.97	88.07 ± 3.00	NS
Range	64.1–94.4	81.4–93		64.1–94.4	80.2–93.8	
MCHC (gm/dL)						
Mean ± SD	32.78 ± 1.54	33.62 ± 1.28	0.001	33.00 ± 1.40	32.56 ± 1.65	NS
Range	29–36	31.4–35.3		29.9–35.3	29–36	
PLT (10^3^/mm^3^)						
Mean ± SD	293.00 ± 57.81	233.77 ± 51.67	0.001	276.77 ± 61.47	309.23 ± 49.38	0.005
Range	180–421	170–370		180–385	206–421	
CRP (gm/L)						
Mean ± SD	8.55 ± 2.53	3.46 ± 0.78	0.001	5.38 ± 1.36	11.71 ± 2.55	0.001
Range	2.6–26.2	2.1–4.5		2.6–9.1	6.4–26.2	
AK155(IL–26) (pg/mL)						
Mean ± SD	31.47 ± 8.06	13.87 ± 2.71	0.001	21.79 ± 4.72	41.15 ± 7.58	0.001
Range	12.1–53	9.9–22		12.1–34	23–53	

T-independent sample *t*-test was used to compare data that follow the normal distribution, and data were presented as Mean ± SD. x^2^: Chi-square test for Number (%) or Fisher’s exact test was used to compare between data that do not follow the normal distribution and presented as Median and Interquartile Range (IQR). A *p*-value < 0.5 was considered statistically significant. [COPD: chronic obstructive pulmonary disease, BMI: body mass index, PCO_2_: partial pressure of CO_2_, PO_2_: partial pressure of O_2_, HCO_3_: bicarbonate, S O_2_%: O_2_ saturation, FEV1%: Forced Expiratory Volume in the first second of expiration, FVC%: Forced Vital Capacity, FEV1/FVC: Forced Expiratory Volume in the first second of expiration/Forced Vital Capacity, VC%: Vital Capacity, FEF%: Forced Expiratory Flow Rate, WBCs: White Blood Cells, RBCs: Red Blood Cells, Hb: Hemoglobin, HCT: Hematocrit, MCV: Mean Corpuscular Volume, MCHC: Mean Corpuscular Hemoglobin Concentration, PLT: platelets, CRP: C-Reactive Protein, AK155(IL-26): Interleukin-26, NS: non-significant].

**Table 2:** *TLR9 rs5743836* SNP in the studied groups.

TLR–9	Group (n)			COPD Group (n)		
rs5743836SNP	COPD (96)	Control (48)	OR (95% CI)	Exacerbation (48)	Stable (48)	OR (95% CI)
*Genotypes*						
TT	31 (32.1%)	32 (66.7%)	Reference	11 (22.9%)	20 (41.7%)	Reference
TC	50 (52.1%)	16 (33.3%)	3.22 (1.5–6.82) *	27 (56.3%)	23 (47.9%)	2.13 (0.84–5.37)
CC	15 (15.6%)	0 (0.0%)	31.98 (1.8–57.7) *	10 (20.8%)	5 (10.4%)	3.64 (0.9–13.36) *
*Alleles frequency*						
T	112 (58.3%)	80 (83.3%)	Reference	49 (51.0%)	63 (65.6%)	Reference
C	80 (41.7%)	16 (16.7%)	3.57 (1.94–6.56) *	47 (49.0%)	33 (34.4%)	1.83 (1.02–3.27) *
*Dominant model*						
TT	31 (32.1%)	32 (66.7%)	Reference	11 (22.9%)	20 (41.7%)	Reference
TC + CC	65 (67.7%)	16 (33.3%)	4.19 (2.01–8.76) *	37 (77.1%)	28 (58.3%)	2.40 (0.99–5.82) *
*Recessive model*						
TT + TC	81 (84.4%)	48 (100.0%)	Reference	38 (79.2%)	43 (89.6%)	Reference
CC	15 (15.6%)	0 (0.0%)	18.45 (1.1–35.3) *	10 (20.8%)	5 (10.4%)	2.26 (0.71–7.2)

The odds ratio (OR) of genotypes between groups was assessed using logistic regression analysis. * A *p*-value is significant < 0.05. [*TLR9*: Toll-Like Receptor-9, COPD: chronic obstructive pulmonary disease, OR: odds ratio, CI: Confidence Interval, NS: non-significant].

**Table 4:** Correlation among serum levels of AK155(IL-26) with different parameters in the exacerbated (n = 48) and stable COPD patients (n = 48).

	AK155(IL−26) (pg/mL) in the COPD Group			
	Exacerbated (n = 48)		Stable (n = 48)	
**Variables**	**r-Value**	***p*-Value**	**r-Value**	***p*-Value**
Age (years)	−0.155	NS	−0.07	NS
Smoking index	−0.124	NS	0.172	NS
BMI [kg/m^2^]	0.790	0.001	0.974	0.001
pH	−0.164	NS	−0.111	NS
PCO_2_ (mmHg)	0.268	NS	0.126	NS
PO_2_ (mmHg)	−0.188	NS	0.058	NS
HCO_3_ (mmol/L)	0.222	NS	0.027	NS
S O_2_%	−0.181	NS	0.005	NS
FEV1%	−0.915	0.001	−0.873	0.001
FVC%	0.008	NS	−0.190	NS
FEV1/FVC	−0.731	0.001	−0.87	0.001
VC%	0.061	NS	0.326	0.024
FEF%	−0.066	NS	−0.062	NS
WBCs (10^3^/mm^3^)	0.151	NS	0.23	NS
RBCs (10^3^/mm^3^)	0.029	NS	0.116	NS
Hb (gm/dL)	0.034	NS	0.269	NS
HCT%	0.052	NS	0.180	NS
MCV (µm3)	−0.002	NS	−0.151	NS
MCHC (gm/dL)	0.094	NS	−0.112	NS
PLT (10^3^/mm^3^)	0.238	NS	−0.273	NS
CRP (gm/L)	0.755	0.001	0.963	0.001

Pearson’s correlation coefficient (r). [COPD: chronic obstructive pulmonary disease, BMI: body mass index, PCO_2_:, Partial Pressure of CO_2_, PO_2_: Partial Pressure of O_2_, HCO_3_: bicarbonate, S O_2_: O_2_ saturation, FEV1%: Forced Expiratory Volume in the first second of expiration, FVC%: Forced Vital Capacity, FEV1/FVC: Forced Expiratory Volume/Forced Vital Capacity, VC%: Vital Capacity, FEF%: Forced Expiratory Flow Rate, WBCs: White Blood Cells, RBCs: Red Blood Cells, Hb: hemoglobin, HCT: hematocrit, MCV: Mean Corpuscular Volume, MCHC: Mean Corpuscular Hemoglobin Concentration, PLT: platelets, CRP: C-Reactive Protein, AK155(IL-26): Interleukin-26, NS: non-significant].

## Data Availability

The original contributions presented in the study are included in the manuscript. Further inquiries can be provided by the corresponding author upon request.

## Abbreviations

AK155(IL-26)	Interleukin-26.
BMI	Body mass index.
CD	Cluster of Differentiation.
COPD	Chronic obstructive pulmonary disease.
CpG	Cytosine-phosphate guanine.
CRP	C-Reactive Protein.
ECOPD	Exacerbated chronic obstructive pulmonary disease.
FEF%	Forced Expiratory Flow Rate.
FEV1/FVC	Forced Expiratory Volume in the first second of expiration/Forced Vital Capacity.
FEV1%	Forced Expiratory Volume in the first second of expiration.
FVC%	Forced Vital Capacity.
GWAS	Genome-Wide Association Studies.
Hb	Hemoglobin.
HCO3	Bicarbonate.
HCT	Hematocrit.
JAK	Janus kinase.
JAK-STAT	Janus Kinase Signal Transducer Activator of Transcription.
MCHC	Mean Corpuscular Hemoglobin Concentration.
MCV	Mean Corpuscular Volume.
MyD88	Myeloid differentiation primary response gene 88.
NF-_k_B	Nuclear factor Kappa Beta.
NK	Natural Killer.
PCO_2_	Partial Pressure of CO_2_.
PDCs	Plasmacytoid dendritic cells.
PLT	Platelets.
PO_2_	Partial Pressure of O_2_.
RBCs	Red Blood Cells.
RT-PCR	Reverse Transcriptase-Polymerase Chain Reaction.
SI	Smoking index.
S O_2_	O_2_ saturation.
SARS-CoV-2	Severe Acute Respiratory Syndrome-Corona Virus-2.
SNP	Single-Nucleotide Polymorphism.
Th-17	T helper-17.
TLR9	Toll Like Receptor-9.
TyK	Tyrosine Kinase.
VC%	Vital Capacity.
